# Global Microbiome: Core and Unique Signatures Across Diverse Populations

**DOI:** 10.3390/ijms27041776

**Published:** 2026-02-12

**Authors:** Sherri Huang, Diptaraj S. Chaudhari, Rohit Shukla, Pushti Kanani, Rola S. Zeidan, Yi Lin, Wesley Burrow, Robert T. Mankowski, Shalini Jain, Hariom Yadav

**Affiliations:** 1 Kaiser Permanente San Leandro Medical Center, San Leandro, CA 94577, USA; 2 USF Center for Microbiome Research, Microbiomes Institute, Tampa, FL 33612, USA; 3 Department of Physiology, Wayne State University School of Medicine, Detroit, MI 48301, USA; 4 Department of Neurosurgery, Brain and Spine, University of South Florida, Tampa, FL 33606, USA; 5 Department of Physiology and Aging, College of Medicine, University of Florida, Gainesville, FL 32610, USA; rzeidan@ufl.edu (R.S.Z.);; 6 Department of Health Outcomes and Biomedical Informatics, College of Medicine, University of Florida, Gainesville, FL 32611, USA; 7 Dr. Kiran C. Patel College of Allopathic Medicine, Nova Southeastern University, Fort Lauderdale, FL 33328, USA; 8 Division of Gerontology, Geriatrics and Palliative Care, Department of Medicine, University of Alabama at Birmingham, Birmingham, AL 35294, USA

**Keywords:** microbiome, world, global, core, unique, enterotype, aging

## Abstract

Earlier analyses evaluating patterns of gut microbiota in individuals from different geographies and age groups are heterogeneous in methodology, precluding broader conclusions about the relationship between the gut microbiome and geographic region, age, and clinical health. Here, we systematically conducted a meta-analysis of 16s rRNA gut microbiome sequencing data representing 10,878 samples across North America, Europe, Africa, Asia and Oceania. Our analysis included 27 countries and three age groups (neonate to age 17, or AG01; ages 18 to 64, or AG02; 65 and above, or AG03). We identified that Firmicutes, Bacteriodetes, and Proteobacteria constitute core phyla across geographic regions. A differing predominance of top families alongside core family *Lachnospiracaeae* across regions comprised unique microbiome signatures. Countries also differed in their relative abundances of *Bifidobacterium*, *Faecalibacterium*, *Lactobacillus* and *Bacteroides.* We found in our age analyses that Proteobacteria and Actinobacteria were the most abundant phyla in AG01, and Actinobacteria abundance declined across all continents with increasing age. The relative abundance of Bacteriodetes increased between AG01 and AG02. Enrichment of asthma-associated *Enterobacterieaceae* in AG01 was highest for North America, followed by Europe and then in Asia. We discuss the correlation of these gut microbial patterns in the context of dietary patterns, populations health, clinical health trends, and healthy aging.

## 1. Introduction

In the past 15 years, patterns of human gut microbiota composition have been investigated in the epidemiology and outcomes of the spectrum of chronic disease, including cardiovascular disease, diabetes, inflammatory bowel disease, cancers, and pediatric atopic conditions [1,2,3,4,5,6,7,8]. The human gut microbiome is subject to influences from dietary habits to environmental exposures which vary across geographic regions. The literature has evaluated the impact of environmental exposures across different regions on the gut microbiome. For example, exposure to heavy metals such as arsenic, lead, and cadmium and air pollution have been linked to altered gut composition; a study on two communities in Southern Nepal found that consumption of arsenic-contaminated water resulted in a decrease in gut commensal bacteria, while another study of healthy individuals from two separate villages in China found that chronic exposure to arsenic, cadmium, copper, lead and zinc lead to a decrease in *Prevotella* [9,10]. These environmental associations are replicated in other geographic regions; exposure to traffic-related air pollution in young adults in Southern California also led to gut dysbiosis [11]. Dietary influences are also seminal in gut microbiota composition [12,13,14,15,16].

As a dynamic entity, the gut microbiome is poised to potentially explain the rates of chronic diseases across geographic regions and age groups, specifically: (1) What are the core and unique signatures of gut microbiome patterns across the world’s geographic regions and how do they correlate with disease incidence? (2) How do these patterns correlate with age-related pathologies? Up until now, there have been limited studies addressing these questions. Existing studies on microbiome patterns reveal differences across geography and age; however, conclusions about global patterns are limited by the heterogeneous regions, sample sizes, and microbiome types studied [17,18,19,20,21,22,23,24,25,26]. In recent years, microbiome hallmarks of healthy aging have emerged; however, whether these are replicated or unique across geographic regions remains to be studied [27,28,29,30]. In addition to understanding the impact of the dynamic microbiome on disease incidence across global regions and ages, understanding these differences will help us comprehend global biodiversity and complex relationships between culture, microbiota composition, and health status at the population level. A larger study is therefore needed to understand the relationships between gut microbiome patterns across countries and environmental exposures, dietary habits, and health consequences. We performed a meta-analysis of gut microbiome datasets from North America, Europe, Africa, Asia and Oceania across age groups to evaluate the identities and distribution of gut microbiota across geography and age.

## 2. Results

### 2.1. Literature Screening, Metadata Screening and Final Analysis Input

We queried and screened publications on PubMed to generate a list of studies with datasets representing 16s rRNA samples of human healthy control groups from stool collections. The number of publications resulting from our initial PubMed literature screen and from the secondary literature screen for each continent and country is shown in Table 1. The initial and final numbers of metadata included in the analyses by continent are shown in Table 2, along with the initial number of metadata by country, where available. In total, 19,464 metadata records resulted from the second metadata screen, of which 10,878 underwent further quality control to be included in the final phylogenetics analyses. Of the 10,878, North America comprised 2902; Asia comprised 2433, Africa comprised 454; Europe comprised 5004; and Oceania comprised 87 metadata records.

### 2.2. Core Microbiome Signatures Across Continents

#### 2.2.1. Diversity Indices

**Figure 1:**
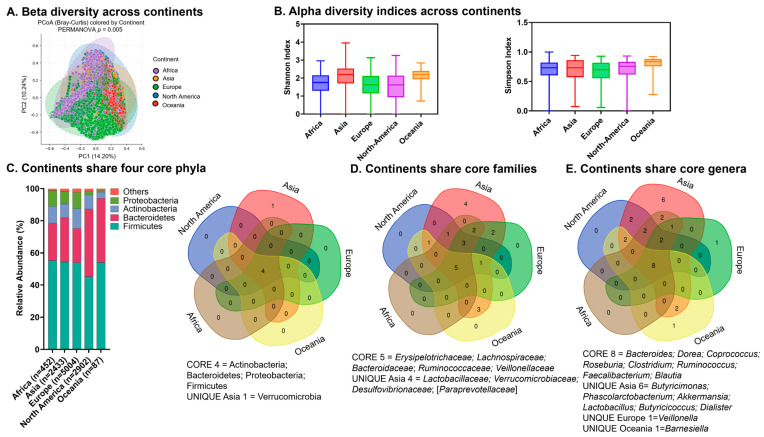
The gut harbors a core phyla microbiome signature in the representative world population at the continent level. (**A**) Beta diversity across continents; (**B**) alpha diversity indices across continents; (**C**) relative abundances of core phyla across continents (**left**) and Venn diagram analysis showing core phyla (**right**); (**D**) Venn diagram analysis showing core families across continents; (**E**) Venn diagram analysis showing core genera across continents.

The gut microbiome harbors a core microbiome signature within the representative world population. Beta diversity among the five included continents was low, with most data points centered between −5 and 5, suggesting similar compositions across continental communities (Figure 1A). Shannon and Simpson indices were comparable in all continents, suggesting similar levels of overall species diversity (Figure 1B).

#### 2.2.2. Core Microbiome

All continents shared four core phyla, which for each continent altogether comprised over 98% of the total relative abundance (Figure 1C). These were Firmicutes, Bacteroidetes, Proteobacteria, and Actinobacteria, with Firmicutes and Bacteriodetes being the most dominant among all continents; consistently, the Venn diagram analysis shows these as four shared phyla (Figure 1C). The Venn diagram analysis shows the five top shared families across continents including three in the dominant phyla—*Bacteroidaceae* (phylum Bacteriodetes), *Ruminococcaceae* (phylum Firmicutes) and *Veillonellaceae* (phylum Firmicutes)—along with *Erysipelotrichaeceae* and *Lachnospiraceae* (Figure 1D). Venn diagram analysis shows the eight top shared genera across continents, including *Bacteroides* (Figure 1E).

We next calculated the relative abundance of Actinobacteria compared to Proteobacteria, also known as the A/P ratio. Higher ratios indicate a higher relative abundance of Actinobacteria, while lower ratios (below 1) indicate a higher relative abundance of Proteobacteria. North America demonstrated the lowest ratio, while Europe demonstrated the highest A/P ratio (Table 3).

### 2.3. Core Microbiome Signatures Across Countries

#### 2.3.1. Diversity Indices

**Figure 2:**
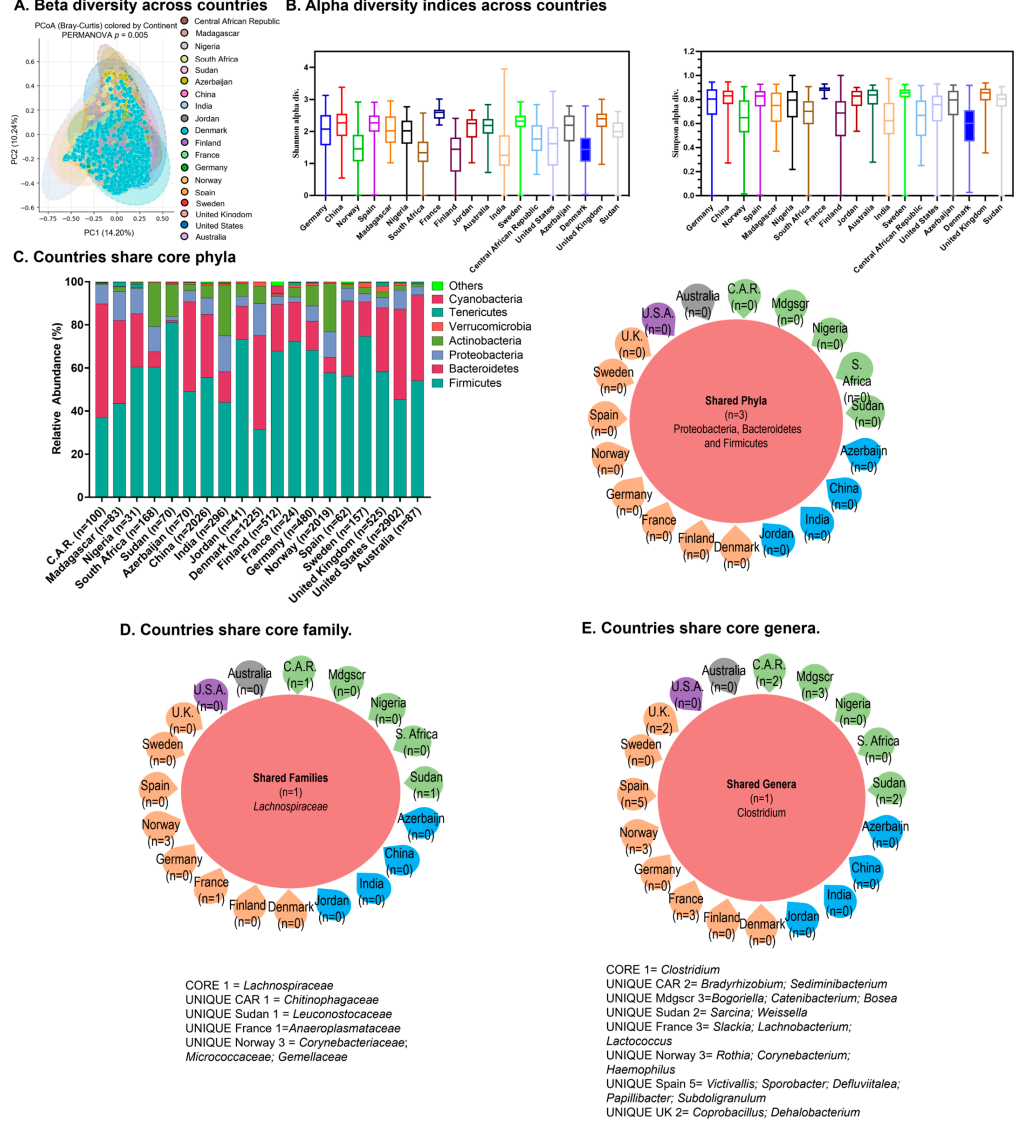
The gut harbors core microbiome signatures in the representative world population at the country level. (**A**) Beta diversity across countries; (**B**) alpha diversity indices across countries; (**C**) relative abundances of core phyla across countries (**left**) and Venn diagram analysis showing core phyla across countries (**right**). (**D**) Venn diagram analysis showing core families across countries and (**E**) Venn diagram analysis showing core genera across countries.

Beta diversity across countries showed most values centered around 5 and −5 again (Figure 2A). Alpha diversity as measured by the Shannon index was more varied across countries but indices were similar when calculated by Simpson index (Figure 2B).

#### 2.3.2. Core Microbiome

Our analysis also revealed core phyla at the country level: Firmicutes, Bacteroidetes, and Proteobacteria altogether comprised at least 70% of total relative abundance (Figure 2C, left). Consistent with the relative abundances, Venn diagram analysis shows three shared phyla identified as Proteobacteria, Bacteroidetes and Firmicutes across countries (Figure 2C, right). For the majority of evaluated countries, Firmicutes made up the largest proportion. The exceptions were Central African Republic, Denmark, and the United States, for which Bacteriodetes made up the largest proportion (Figure 2C, left). For the majority of countries, Proteobacteria was a dominant phylum comprising the largest proportion after Firmicutes and Bacteriodetes. However, Actinobacteria made up the largest proportion after Firmicutes and Bacteriodetes for South Africa, Sudan, India, Jordan, France, Germany and Norway.

*Lachnospiracaeae* represented a top family across the majority of countries (Figure 2D). Consistent with this, the Venn diagram analysis shows one shared top family across countries (*Lachnospiracaeae*). At the genera level, Others comprised the largest abundance for the majority of countries, with the exception of the Central African Republic for which *Prevotella* predominated, and Denmark, for which *Bacteroides* predominated (Figure 2D). The Venn diagram shows *Clostridium* as a shared genus (Figure 2E).

### 2.4. Unique Microbiome Taxonomies Across Continents

#### 2.4.1. Families

**Figure 3:**
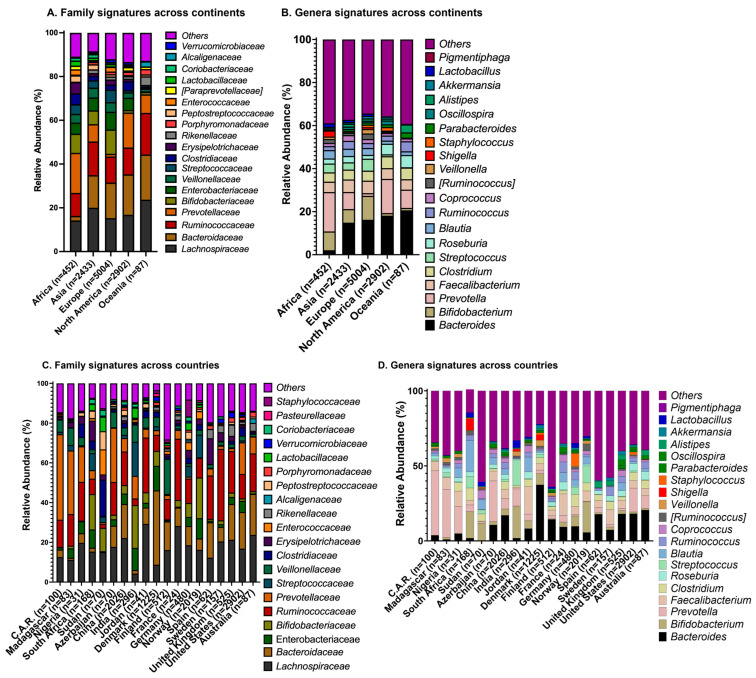
There are unique microbiome signatures for continents and countries. (**A**) Relative abundances of bacterial families across continents and (**B**) relative abundances of bacterial genera across continents. (**C**) Relative abundances of bacterial families across countries; (**D**) relative abundances of bacterial genera across countries.

The distribution of the top taxonomies differed across continents and countries, suggesting unique microbiome signatures across geography (Figure 3). *Lachnospiracaeae* and *Bacteriodaceae* constituted the two largest phylogenetic families in Europe, North America, and Oceania, while *Lachnospiracaeae* and *Ruminococcaceae* were the two largest families in Asia (Figure 3A). *Bacteriodaceae* made up the largest family for Europe and North America; *Lachnospiracaeae* made up the largest phylogenetic family for Africa, Asia, and Oceania. *Prevotellaceae* and *Lachnospiracaeae* made up the two largest families for Africa, followed by *Ruminococcaceae* (Figure 3A).

#### 2.4.2. Genera

With the exception of Africa, Others and *Bacteroides* constituted the largest proportion of phylogenetic genera groups across all continents (Figure 3B). Asia, North America and Oceania shared similar top three frequencies; Others, *Bacteroides* and *Prevotella* represented the three largest genera proportion in these regions. In Africa, Others and *Prevotella* accounted for the largest genus proportions, while *Bifidobacterium* was the third largest genus proportion in Africa and Europe.

### 2.5. Unique Microbiome Taxonomies Across Countries

#### 2.5.1. Families

*Lachnospiraceae*, *Ruminococcaceae*, and *Bacteriodaceae* were prominent among the top phylogenetic families across the countries assessed (Figure 3C). In 12 out of 19 countries assessed, *Lachnospiracaeae* was one of the top two families. In 8 of 19 countries, *Ruminococcaceae* was one of the top two families. *Bacteriodaceae* was one of the top two families in 5 out of 19 countries.

Other and *Veillonella* were the top two families in Finland. *Bifidobacterium* was among the top two families in India and South Africa. *Prevotellaceae* was the top family in Madagascar, the Central African Republic and Azerbaijan.

#### 2.5.2. Genera

Others constituted the largest proportion of genus in all countries except for Denmark, where *Bacteriodes* was the largest genus, and the Central African Republic where *Prevotella* was the largest genus (Figure 3D). *Bacteriodes* was the second largest proportion in seven countries. Among the countries assessed, India had the highest relative proportion of *Lactobacillus*.

### 2.6. Enterotypes and LefSe Analyses Across Continents and Countries

Enterotypes analysis using Partitioning Around Medoids (PAM) clustering of genera with continents revealed that North America, Europe and Asia were driven by *Prevotella*. Oceania is driven by Bacteroides, while Africa is driven by Others (Figure S1A). The same distance analysis approach on countries revealed clustering of all analyzed countries with the exception of Sudan and India; the Central African Republic, Madagascar, Nigeria, South Africa, Azerbaijan, China, Jordan, Denmark, Finland, France, Germany, Norway, Spain, Sweden, the United Kingdom, the United States and Australia are driven by the genera *Streptococcus*, *Parabacteroides*, *Faecalibacterium*, and *Akkermansia* (Figure S1B). India is driven by *Akkermansia,* while Sudan is driven by *Alistipes*.

**Figure 4:**
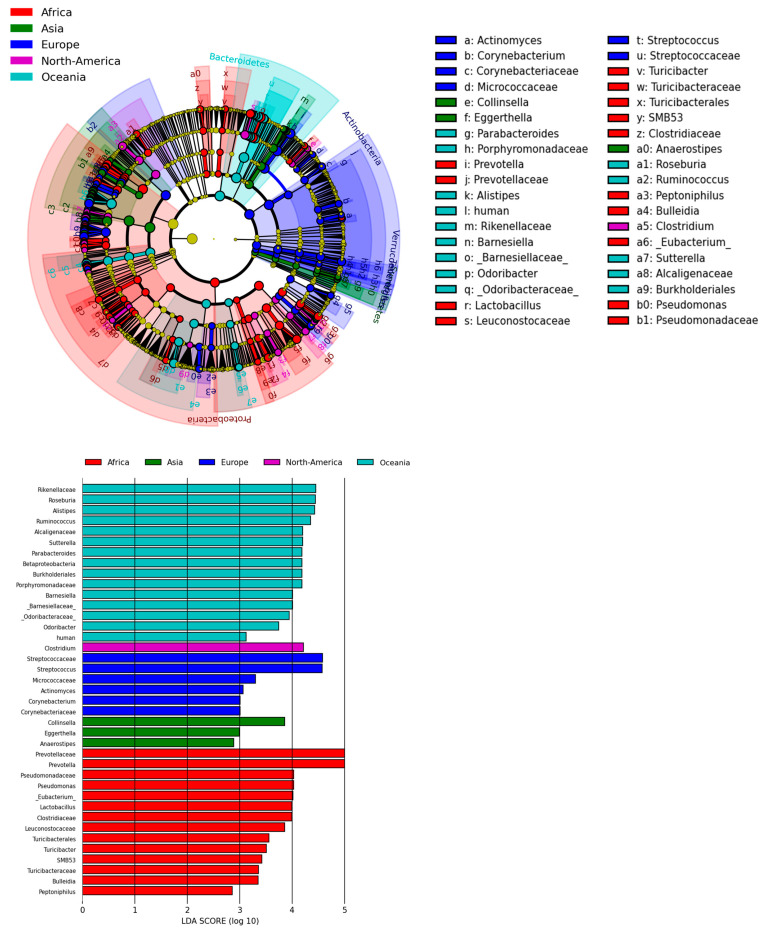
LEfSe analysis shows unique features across continents. LEfSe analysis evaluating clustering via linear discriminant analysis (LDA) effect size revealed patterns consistent with enterotype analysis as well as unique groupings.

Linear discriminant analysis Effect Size (LEfSe) analysis evaluating clustering via the linear discriminant analysis (LDA) effect size revealed patterns consistent with enterotype analysis as well as unique groupings (Figure 4). Based on LEfSe analysis, Africa’s unique signature is most accounted for by the *Prevotellaceae* family and *Prevotella* genus; *Pseudomonadaecae* and *Pseudomonas* made up the next largest LDA effect size. Asia’s unique signature is most accounted for by the *Collinsella* genus, and then by *Eggerthella*; both are part of phylum Actinomycetota. *Streptococcacacaea* and *Streptococcus* comprised the largest LDA score for Europe for family and genus, respectively. *Clostridium* made up the largest LDA score for North America. The *Rikenellacaea* family and *Roseburia* genus comprised the largest LDA score for Oceania.

### 2.7. Age-Associated Changes in Microbiome

**Figure 5:**
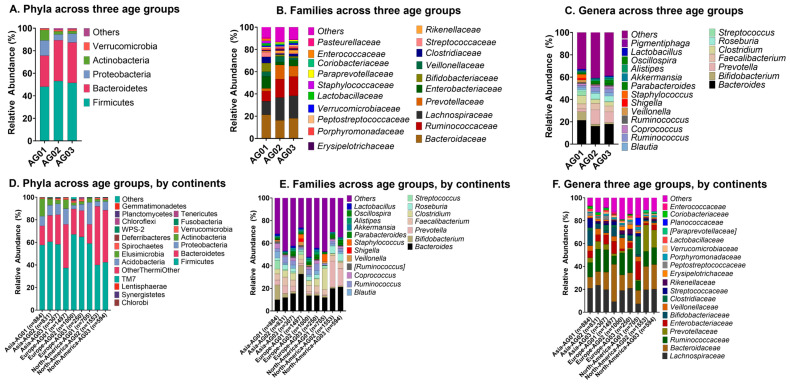
The core microbiome signatures vary according to age. (**A**) Relative abundances of phyla across the three age groups; (**B**) relative abundances of families across the three age groups; (**C**) relative abundances of genera across the three age groups; (**D**) relative abundances of phyla across the three age groups within each continent; (**E**) relative abundances of families across the three age groups within each continent; (**F**) relative abundances of genera across the three age groups within each continent.

We evaluated the microbiome composition across three age groups, defined as AG01 (neonate to age 17; pediatrics), AG02 (age 18 to 64), and AG03 (age ≥ 65). Age has a significant effect on shaping the microbiome composition. Across all three age groups, Firmicutes and Bacteroidetes predominated the relative abundance of gut composition (Figure 5A). Uniquely, Proteobacteria and Actinobacteria comprised the next most common abundance for the AG01 group (Figure 5A).

Firmicutes/Bacteroides (F/B) ratios decreased from AG01 through AG03 (Figure 5A). Proteobacteria and Actinobacteria were most abundant in AG01, with a decreasing Actinobacteria to Proteobacteria ratio as age progressed. The abundances of families *Lachnospiracae* and *Ruminococcaceae* increased from the AG01 through AG03 age groups (Figure 5B). *Shigella* and *Staphylococcus* were the most abundant genera in AG01, while the relative abundances of *Bifidobacterium* and *Streptococcus* decreased across age groups (Figure 5C).

Along with age, geographic location has an impact on shaping the microbiome. The F/B ratio decrease is consistently observed at the geographic level in Asia, Europe and North America (Figure 5D). In both Asia and North America, the relative abundance of Bacteroidetes increases from AG01 to AG02, concurrent with the increase in *Bacteroides* family (Figure 5E). Conversely, in Europe the abundance of Bacteroidetes decreases from the pediatric to adult age groups. Actinobacteria, on the other hand, decreases across AG01 to AG03 on all continents, which is consistent with the decline in the *Bifidobacterium* family across all age groups. Furthermore, *Lachnospiracaea* abundances increase with age in both Europe and North America while remaining steady in Asia (Figure 5F).

## 3. Discussion

Our analysis identified core signatures across geographic regions at the phylum level, with Firmicutes, Bacteriodetes, and Proteobacteria being shared among the five continents we evaluated, consistent with prior analyses [17,31,32]. We found that North America demonstrated the lowest Actinobacteria-to-Proteobacteria (A/P) ratio, while Europe demonstrated the highest A/P ratio. *Lachnospiracaeae* was a core family across Europe, North America, Oceania and Asia; however, the differing predominance of top families alongside *Lachnospiracaeae* across regions composed unique microbiome signatures. Countries also differed in their relative abundances of *Bifidobacterium*, *Faecalibacterium*, *Lactobacillus* and *Bacteroides.* In our age analyses, Proteobacteria and Actinobacteria were the most abundant phyla in AG01; with increasing age, Actinobacteria abundance declined for all continents. The relative abundance of Bacteriodetes increased between AG01 and AG02, concurrent with the increase in relative abundance of family *Prevotellaceae*. *Enterobacterieaceae* was most abundant for North America in AG01, followed by Europe and then in Asia. Finally, the abundance of core family *Lachnospiracaeae* increased with age in Europe and North America but remained steady in Asia.

### 3.1. Core and Unique Signatures and Clinical Correlations Across Geography

A core microbiome shared across geographic regions suggests that microbial constituents may have coevolved and play important roles in responding to environmental pressures such as diet. Members of Firmicutes, for example, include those in class *Bacilli* and *Sphingobacteria*, the latter of which includes species shown to be a critical symbiont in the evolution of single-cellular to multi-cellular organisms, suggesting early evolutionary roots as a potential contribution to gut composition [33]. The class *Bacilli* includes genera *Lactobacillus* whose members play critical roles in metabolism as well as immune regulation and protection from pathogenic microorganisms [34,35]. Members of Firmicutes and Bacteroidetes include commensal microbiota that maintain gut homeostasis, and alteration in the Firmicutes-to-Bacteriodetes ratio is linked to clinical pathogenesis [36,37,38]. Similarly, Proteobacteria dysbiosis is thought to present a risk factor for multiple diseases [39,40].

Firmicutes and Bacteriodetes abundances are also well-documented biomarkers of diet and may explain whether a geographic region exhibited higher relative abundance of Firmicutes versus Bacteroidetes in our analyses [15,16,41,42]. Diets heavy in animal-based protein and saturated fats, for example, have been associated with increased counts of anaerobic species belonging to *Bacteroides* [13,14,16]. Similarly, the comparative intake of fiber-rich foods to meat in a diet in turn may reflect in the Actinobacteria-to-Proteobacteria (A/P) ratio. The relative A/P ratios between North America (lowest A/P ratio), Europe (high A/P), and Asia (intermediate A/P ratio) may therefore be explained by patterns in intake of meat to fruits and vegetables in constituent countries [43,44].

*Lachnospiraceae* members modulate various beneficial and pathogenic processes in the human gut, potentially explaining why this family comprises a core taxonomic signature across most countries [45,46,47]. However, in our study, the differing predominance of top families alongside *Lachnospiracaeae* across regions composed unique microbiome signatures. *Lachnospiracaee* and *Bacteriodaceae* comprised the two largest families in Europe, North America, and Oceania, whereas *Lachnospiracaeae* and *Ruminococcaceae* comprised the two largest families in Asia. Patterns of diet across regions may again contribute to these differences. For example, high levels of animal-based protein intake may explain the dominant presence of *Bacteriodaceae* for Europe and North America, while relatively higher levels of intake of vegetables in China may explain the predominance of *Ruminococcaceae* in our analysis of Asian countries [13,14,16,41,48]. Similarly, our LEfSe and community composition analyses revealed the predominance of *Prevotella* in datasets from Africa; *Prevotella* is associated with a plant-based diet, consistent with dietary patterns studied in African communities [43].

Unique microbiome signatures may manifest clinically and correlate with disease epidemiology across countries. Species that negatively associate with and may have a protective effect on the development of colorectal cancer (CRC) include those belonging to *Bifidobacterium*, *Faecalibacterium*, *Lactobacillus*, and *Prevotella* [49,50,51,52]. We found that most countries in Asia and Africa demonstrated high composite relative abundances of *Bifidobacterium*, *Faecalibacterium* and *Prevotella*. The Central African Republic demonstrated the highest composite relative abundance of these genera, followed by Madagascar, Nigeria, Azerbaijan, India, Jordan, and South Africa. Members of *Bacteroides*, in contrast, are positively associated with CRC development. Coincidentally, countries with the highest composite *Bifidobacterium*, *Faecalibacterium* and *Prevotella* also demonstrated the lowest *Bacteroides* abundances (i.e., Madagascar, India, South Africa, Central African Republic, Nigeria, and Norway). Consistent with documented CRC incidence across countries, countries with the highest composite *Bifidobacterium*, *Faecalibacterium*, and *Prevotella* and the lowest *Bacteroides* abundances were overall correlated with lower CRC incidence [53].

### 3.2. Age-Related Gut Microbiome Changes

Our analysis demonstrated that Proteobacteria and Actinobacteria were the most abundant phyla in AG01. Proteobacteria comprises the core microbiome of human maternal milk [54]. As the incidence of breastfed infants < 6 months around the world is around 88%, the enrichment of Proteobacteria in our AG01 analyses may therefore reflect the predominance of neonate stool samples in our pediatric population [55]. Our study found that AG01 also exhibited the highest relative abundance of *Bifidobacterium*, whose enrichment is promoted in breastfeeding [56].

We found that the relative abundance of Bacteriodetes increased between AG01 and AG02, concurrent with increase in relative abundances of the genus *Prevotella*. Consistent with this observation, the Firmicutes/Bacteroidetes (F/B) ratios decreased from AG01 through AG03. Lower proportions of Bacteriodetes and increasing F/B ratios are associated with obesity, whereas *Prevotella* enrichment is found in high-plant, low-fat diet states [16,36,41,42,43]. As the incidence of inflammatory conditions such as obesity, type 2 diabetes, and cardiovascular disease increases with age, this decrease in F/B ratio with age may be surprising. However, this may be more due to the expansion of diet from the neonatal period, such as the inclusion of plants and other fiber-containing solid foods with increasing age. This is consistent with the increase in *Ruminococcaceae* across age groups. Future studies which evaluate *Prevotella* alterations arising from the initiation of solid foods, around 4 or 6 months of infancy as advised by the American Academy of Pediatrics, may enlighten gut microbiome changes throughout infancy.

### 3.3. Age-Related Gut Microbiome Patterns Across Geography

Clinical conditions such as asthma and dementia are well-characterized as exhibiting age-specificity, i.e., pertaining to pediatrics or ages < 18 (AG01) versus ages > 18 (AG02 and AG03). Consistent with studies showing the inverse correlation of Actinobacteria with dementia, Actinobacteria shows a consistent decline across all continents with increasing age [57]. Asia demonstrated the greatest decrease in Actinobacteria across age groups, consistent with its high age-standardized incidence rates (ASIRs) of Alzheimer’s and other dementias [58]. Asia and North America demonstrate the highest ASIR of Alzheimer’s and other dementias, which may correlate with their increasing abundances of Bacteroides from AG01 to AG02.

The family *Enterobacterieaceae* is one of the most studied taxonomies with respect to pediatric asthma [59,60,61]. In our AG01 analyses, *Enterobacterieaceae* was most abundant for North America, followed by Europe and then in Asia. A study of the age-standardized disability-adjusted life years (DALYs) rate per 100,000 children in 2019 showed that this pattern correlates with the global pediatric asthma burden, in which the United States ranked highest and Asia ranked lowest [62].

The abundance of *Lachnospiracaeae* increases with age in both Europe and North America while remaining steady in Asia. *Lachnospiracaeae* genera including *Roseburia* and *Blautia* are implicated in gut inflammation and atherosclerosis. While the changes in *Lachnospiracaea* with age is consistent with the increase in obesity, diabetes, and cardiovascular disease rates with age in North America and Europe, it is not immediately clear how the specific age-related signature for Asia correlates clinically. Importantly, the interactions between microbiota communities, such as the predominance of *Ruminococcaceae* for Asia, may modulate or lead to increased complexity in clinical correlations across geography.

Healthy aging is well-characterized in the literature and shown to be modulated by gut microbiota compositions that slow inflammatory processes [27,28,29,30]. With aging, pathobionts such as *Akkermansia* and *Roseburia* species increase in the gut, while anti-inflammatory short-chained fatty acid producers decrease. Consistent with these studies, our analyses revealed that across all geographies and age groups, relative abundances of *Roseburia* and *Akkermansia* increased.

## 4. Materials and Methods

### 4.1. Publication Screening

In the initial screen, the database PubMed was screened for all studies on the human gut microbiome across different global regions using the search terms “[region] human microbiome and 16s rRNA gene and age”, and studies were then further screened to include only those utilizing stool samples (Table 4). All studies through 31 July 2022 were included in this screen. Next, papers were included or excluded for future meta-analysis with the inclusion criteria 16s rRNA, human, gut, and 1st/baseline datapoint, and excluded with the following criteria: metagenomics, non-human, non-gut, and unavailable accessions/unavailable raw data files. Publications resulting from this final screen and for which 16s rRNA data was included in our phylogenetic analyses are provided in Supplementary Table S1: Source Publications.

### 4.2. Metadata Screening

To ensure the inclusion of only metadata representing stool samples from healthy individuals, we first screened the sequencing data from each publication. Non-stool samples and those lacking information relating to the geographic location were excluded. Additionally, samples from participants in intervention or disease groups were omitted. For publications with unknown variables, such as unlisted geographic location, age, or intervention versus disease groups, we reached out to the authors to obtain further information.

### 4.3. Analysis

#### 4.3.1. Sequencing Data Processing and Analysis

Sequencing data for metadata that passed the metadata screening step were downloaded. Data from each publication was processed through the QIIME2 platform (v2021.4) [63]. To avoid ambiguity associated with the DADA2 error model across different sequencing runs, each dataset was analyzed independently. We denoised the raw reads separately for each study using the same QIIME2 pipeline and parameters within each study for ensuring consistency and avoiding the cross-study inference of the analysis parameters. The data were initially imported and demultiplexed, generating interactive quality plots. The interactive quality plots were used to determine the truncation length for forward sequences and reverse sequences (for paired analyses). Utilizing QIIME2’s DADA2 plugin, the sequencing reads were filtered and denoised, resulting in a feature table and representative sequences. The comma-separated value (CSV) files containing the taxonomic information and abundance of taxa from all the datasets were then merged together for the comparative analysis.

Amplicon sequence variants (ASVs) were defined at the study level rather than jointly across datasets. To further account for technical variation, analyses were primarily conducted using relative abundance and the presence–absence of the bacterial-taxa-based beta diversity measures, which are less sensitive to sequencing depth and platform-specific biases. Differences in primer regions and sequencing platforms were addressed by restricting taxonomic comparisons to higher taxonomic ranks, which are genus and phyla, so there is minimal or no effect of these parameters. DNA extraction methods were evaluated qualitatively based on available metadata but were not explicitly corrected, due to incomplete reporting across different studies. Negative and positive controls were not uniformly available across the included studies and were therefore not incorporated into the meta-analysis. However, quality filtering and denoising steps inherent to the QIIME2 pipeline were applied to all datasets to reduce the impact of sequencing artifacts and contaminants.

Beta diversity was determined and other statistical analysis was performed using R packages including Phyloseq, corrplot, vegan and Microbiome [64,65,66,67]. *p*-values for pairwise comparisons for alpha diversity are provided in Supplementary Tables S2–S5. Additional statistical analyses were performed using GraphPad Prism 10 (GraphPad Software, La Jolla, CA, USA). A web-based tool InteractiVenn (https://www.interactivenn.net/ (accessed on 29 November 2023) was used for the analysis of shared and unique bacterial genera [68].

#### 4.3.2. Enterotype Analysis

Genus abundance data were analyzed to assess sample dissimilarity using the Jensen–Shannon Divergence (JSD) metric (calculated after adding a small pseudocount to handle zero values), as per Keller et al. [69]. The resulting distance matrix was clustered using the Partitioning Around Medoids (PAM) algorithm. The optimal number of clusters was determined by evaluating cluster validity indices, primarily the Calinski–Harabasz (CH) index. Community structure was then visualized through ordination methods; principal component analysis (PCA) was performed on the abundance data, followed by Between-Class Analysis (BCA) to maximize the separation and clearly represent the assigned clusters in a reduced-dimensional space.

## 5. Conclusions

Our large-scale analysis of gut microbiota across geography and age confirmed core phyla found in earlier research and revealed unique signatures within different regions of the world that correlated with diet and colorectal cancer incidence. Moreover, prior to this analysis, few studies have evaluated gut microbiome patterns with age except in the context of healthy aging. By identifying microbiome patterns in age transitions, we revealed potential relationships between gut microbiology and age-related disease. We found that changes in the abundance of Actinobacteria correlated with disease incidence of Alzheimer’s in Asia and North America and that the *Enterobacteriaceae* abundance in AG01 across North America, Europe and Asia correlated with the DALYs for asthma across these continents. These age-related microbiome patterns may provide the foundation for public health institutions in efforts to target age groups to reduce the burden of chronic diseases.

Limitations of this study include a predominance of samples from specific countries within continents along with a predominance of neonate samples in the pediatric age group. That is, several regions were underrepresented due to relatively fewer studies in the literature. While Asia, Europe and North America yielded several thousand metadata, Africa yielded 452 (representing 5 publications) and Australia 87 (representing 4 publications). While we did not evaluate gut microbiome relative abundances in a composite sample that pooled all regions, the relatively limited data for Africa and Australia limits the strength of conclusions for their respective analyses. Our study also does not differentiate effectively between neonate populations, young children, and adolescent age groups. However, our results demonstrate distinct gut microbial patterns from the pediatric to adult ages. Factors such as host genetics, culture-specific practices including specific foods and supplements, environment exposures, and their interplay may also influence gut microbiota patterns. The correlation between gut microbiome patterns and CRC incidence and asthma DALYs warrants mechanistic studies to clarify any causal relationship. Multi-disciplinary analyses through biological, sociological and anthropological lenses will most effectively delineate the confounders for future analyses.

## Figures and Tables

**Table 1:** Publication count from literature screening.

Region	Initial Screen	Secondary Screen
**North America**	**124**	**33**
United States	108	30
Canada	16	3
**Asia**	**129**	**41**
China	92	33
India	12	8
Japan	22	0
Korea	3	0
Malaysia	0	0
Mongolia	0	0
Singapore	0	0
**Africa**	**10**	**5**
**Europe**	**61**	**23**
Finland	14	8
Germany	21	6
Sweden	16	4
Denmark	10	5
**Australia**	**16**	**4**

**Table 2:** Final metadata counts by country.

Region	Initial Metadata Screen	Secondary Metadata Screen	Final Metadata
**North America**	**20,758**	**8827**	**2902**
United States	18,928	7166	
Canada	1830	1661	
**Asia**	**62,737**	**3691**	**2433**
China	60,512	2990	
India	2225	701	
Japan	0	0	
Korea	0	0	
Malaysia	0	0	
Mongolia	0	0	
Singapore	0	0	
**Africa**	**1525**	**790**	**452**
Central African Republic		106	
South Africa		323	
Nigeria		40	
Sudan		115	
Madagascar		92	
Tanzania		60	
Botswana		54	
**Europe**	**14,664**	**6032**	**5004**
Finland	2094	971	
Germany	5630	2504	
Sweden	2809	204	
Denmark	4131	2353	
**Australia**	**984**	**124**	**87**
**Total Metadata**		**19,464**	**10,878**
**Other Countries**		**4198**	
United Kingdom		869	
France		24	
Spain		154	
Norway		2995	
Azerbaijan		96	
Jordan		60	

**Table 3:** Actinobacteria/Proteobacteria ratios.

	Actinobacteria	Proteobacteria	Ratio
Europe	12.43	10.22	1.22
Africa	10.45	9.91	1.05
Asia	8.09	8.55	0.95
Oceania	1.19	3.80	0.31
North America	2.24	8.82	0.25

**Table 4:** Search terms applied in literature screen.

Search Term
United States human microbiome AND 16S rRNA gene AND age
Asian human microbiome AND 16S rRNA gene AND age
China human microbiome AND 16S rRNA gene AND age
India human microbiome AND 16S rRNA gene AND age
Japan human microbiome AND 16S rRNA gene AND age
Korea human microbiome AND 16S rRNA gene AND age
Malaysia human microbiome AND 16S rRNA gene AND age
Mongolia human microbiome AND 16S rRNA gene AND age
Singapore human microbiome AND 16S rRNA gene AND age
Australian human microbiome AND 16S rRNA gene AND age
African human microbiome AND 16S rRNA gene AND age
European human microbiome AND 16S rRNA gene AND age
Finland human microbiome AND 16S rRNA gene AND age
Germany human microbiome AND 16S rRNA gene AND age
Sweden human microbiome AND 16S rRNA gene AND age
Denmark human microbiome AND 16S rRNA gene AND age
Canadian human microbiome AND 16S rRNA gene AND age

## Data Availability

Analyses presented in this paper are derived from data available in the public domain (PubMed). For the list of publications and accession numbers of their corresponding data applied in our analyses, please see Supplementary Table S1.

## Supplementary Materials

The following supporting information can be downloaded at: https://www.mdpi.com/article/10.3390/ijms27041776/s1. The list of literature providing the 16s rRNA data we analyzed in our study can be found in the references list at the end of this manuscript [61,70,71,72,73,74,75,76,77,78,79,80,81,82,83,84,85,86,87,88,89,90,91,92,93,94,95,96,97,98,99,100,101,102,103,104,105,106,107,108,109,110,111,112,113,114,115,116,117,118,119,120,121,122,123,124,125,126,127,128,129,130,131,132,133,134,135,136,137,138,139,140,141,142,143,144,145,146]. It is also provided in the Supplementary Table which includes the accession numbers for each study.

## Abbreviations

The following abbreviations are used in this manuscript:

PAM	Partitioning Around Medoids
LEfSe	Linear discriminant analysis Effect Size
A/P ratio	Actinobacteria-to-Proteobacteria ratio
F/B ratio	Firmicutes-to-Bacteroides ratio
CRC	Colorectal Cancer
ASIR	Age-Standardized Incidence Rate
DALYs	Disability-Adjusted Life Years
