# Supplementary figures and images for: Global Microbiome: Core and Unique Signatures Across Diverse Populations
Source: Int J Mol Sci. 2026 Feb 12;27(4):1776. doi: 10.3390/ijms27041776 (PMC12940378; doi:10.3390/ijms27041776)

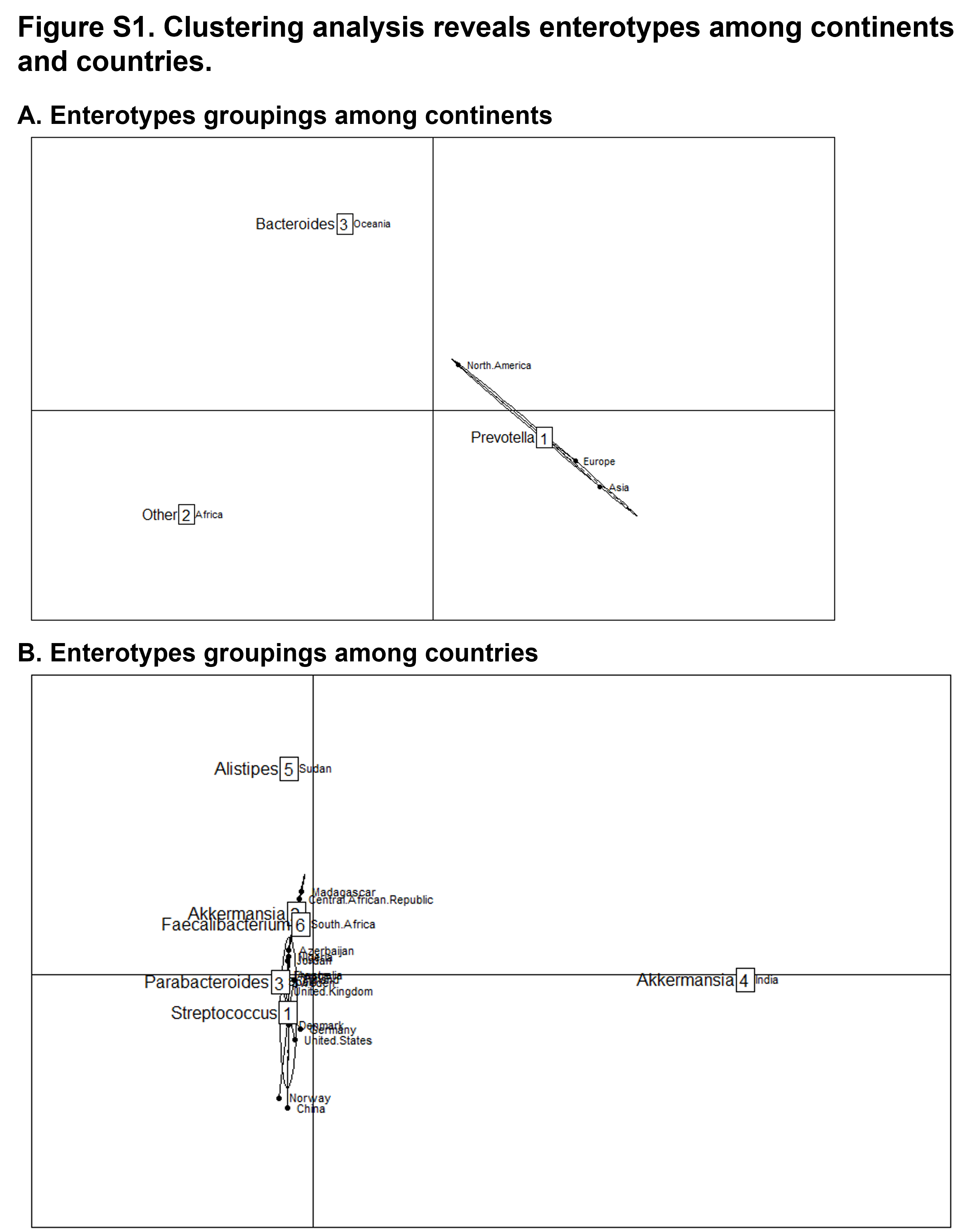

Supplement: Supplementary file 1 [file ijms-27-01776-s001.zip › Figure S1.png]
